# Urine-Derived Induced Pluripotent Stem Cells in Cardiovascular Disease

**DOI:** 10.1155/2020/3563519

**Published:** 2020-01-10

**Authors:** Ping Huang, Yibin Li, M. I. Nasser, Huiming Guo, Huanlei Huang, Mingyi Zhao, Ping Zhu

**Affiliations:** ^1^Guangdong Cardiovascular Institute, Guangdong Provincial People's Hospital, Guangdong Academy of Medical Sciences, Guangzhou, Guangdong 510100, China; ^2^Southern Medical University, Guangzhou, Guangdong 510515, China; ^3^The Third Affiliated Hospital of Southern Medical University, Guangzhou, Guangdong 510000, China; ^4^Department of Pediatrics, The Third Xiangya Hospital of Central South University, Changsha, Hunan 410013, China

## Abstract

Recent studies have demonstrated that stem cells are equipped with the potential to differentiate into various types of cells, including cardiomyocytes. Meanwhile, stem cells are highly promising in curing cardiovascular diseases. However, owing to the ethical challenges posed in stem cell acquisition and the complexity and invasive nature of the method, large-scale expansions and clinical applications in the laboratory have been limited. The current generation of cardiomyocytes is available from diverse sources; urine is one of the promising sources among them. Although advanced research was established in the generation of human urine cells as cardiomyocytes, the reprogramming of urine cells to cardiomyocytes remains unclear. In this context, it is necessary to develop a minimally invasive method to create induced pluripotent stem cells (iPSCs). This review focuses on the latest advances in research on urine-derived iPSCs and their application mechanisms in cardiovascular diseases.

## 1. Introduction

Induced pluripotent stem cells (iPSCs) are well known to be biologically engineered cells derived through the reprogramming of somatic cells. iPSCs were first studied in mice in 2006 by two distinguished researchers Takahashi and Yamanaka [1], and later, a study was conducted in humans [2]. Interestingly, researchers have reported that an injection of iPSCs from somatic cells into the mouse tetraploid blastocysts demonstrated complete reproductive ability, offering evidence of the totipotency of iPSCs [3, 4]. iPSCs, working with the same mechanisms as embryonic stem cells (ESC), can release embryonic stem cell marker genes and have demonstrated potent self-renewal ability, as well as differentiation potential. Moreover, several studies have reported that iPSCs could further differentiate into cardiomyocytes [5–7].

In addition, iPSCs have significant advantages over ESC due to their somatic cell origin, bypassing most ethical objections to ESC technology. Furthermore, they demonstrate unlimited self-renewal capacity and the ability to differentiate into various human cells. These cells deliver an unmatched appropriate source of pluripotent stem cells, promising to establish a new generation for clinical and diagnostic reforms in medicine [8]. Notably, iPSCs are useful not only in new cell therapies but also aid in disease modeling, drug research, and developmental research.

With the continuous development of the reprogramming strategy, human iPSCs were generated from various human tissues, such as keratinocytes [9], blood cells [10], and extramedullary tissues [11], as well as fibroblasts. The reprogramming efficiency of these tissues differed, suggesting that the cellular origin is an essential determinant of the reprogramming efficiency [12, 13].

An ideal cellular source of stem cells should satisfy a few qualities such as accessibility, susceptibility, and universality [14]. As early as 1972, scholars successfully isolated viable cells from fetal urine [15]. In 2011, urine-derived iPSCs (UiPSC) were obtained by reprogramming cells in human urine [16] in particular. The human urinary system is composed of a variety of small tube networks providing a relatively large total surface area compared to the skin. On a daily basis, a massive number of cells are shed from the urinary system, including the ureters and urethra [17]. Moreover, urine production is a crucial physiological process for proper homeostasis in humans. Thus, researchers have hypothesized that the reprogramming of urine cells to cardiomyocytes might be a potential noninvasive strategy in cardiovascular diseases.

**Figure 1:**
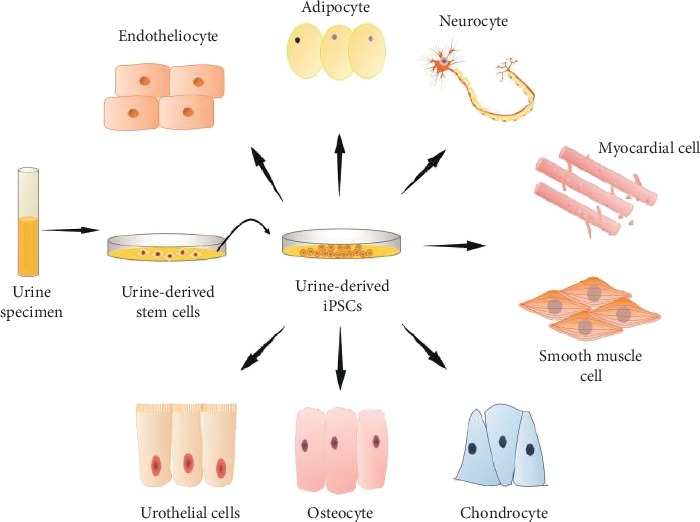
Reprogramming of urine cells and differentiation of UiPSCs.

Additionally, urine-derived iPSCs could be differentiated into a wide range of cells with significant advantages, including urinary epithelial cells, endothelial cells, nerve cells, skeletal myogenic cells, osteoblasts, adipocytes, and chondrocytes [18] (see Figure 1). Since the method to obtain human urine cells is relatively simple, costless, repeatable, and noninvasive, it is particularly suitable for children and the vulnerable elderly population [19, 20]. Therefore, based on these superior features, urine may be the preferred source of iPSCs.

## 2. Introduction of Urine-Derived Cells

In accordance with daily physiology, humans shed approximately 2000 to 7000 cells from the urinary system. These cells are not damaged and can be used for in vitro studies [21]. However, there are controversies regarding the origin of urine cells capable of generating stem cells. According to a recent gene expression profile analysis, urine cells are mainly derived from renal epithelium; however, marker genes in the urothelial cells can also be detected [21]. In recent years, researchers have observed that a subset of urine cells can be easily isolated from urine, namely, urine-derived stem cells (USCs), which can increase steadily expressing stem cell markers and demonstrating the potential for multiple differentiation [22, 23]. Moreover, a recent study indicates that urine-derived stem cells (USCs) could promote mesenchymal stem cell phenotypes, including spindle morphology and expression of cell surface markers CD44, CD73, CD90, CD105, and CD146. Furthermore, USCs do not express hematopoietic stem cell markers such as CD25, CD31, CD34, and CD45. Simultaneously, researchers have reported that USCs in the urine are the main source of the iPSCs [24].

**Figure 2:**
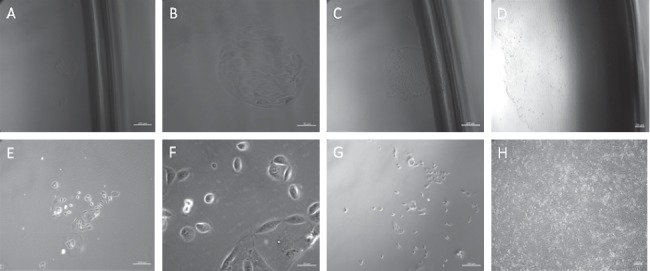
Separation of urine cells. (a)–(d) are type I urine cells ((a) d6, 100×; (b) d6, 400×; (c) d8, 100×; (d) d13, 50×); (e)–(h) are type II urine cells ((e) d6, 100×; (f) d6, 400×; (g) d8, 100×; (h) d13, 50×).

Urine cells are usually isolated in a petri dish coated with gelatin and adhere to the dish after three days. A large number of studies have shown that urine cells might demonstrate two types of cloning [25–27]. According to a previously published method [28], when separating urine cells, we observed that the cell content was higher in urine from women than from men; also, it was easier to separate the urine cells in urine samples derived from men. Considering the day of urine cell separation as day 0, the urine samples from men demonstrated adherent cells on day 3. However, in case of the samples from women, adherence was delayed due to the interference of a large number of epithelial cells. On day 6, due to considerable epithelial cell death, the urine cell clone mass can be observed. In accordance with previous literature, there are two types of clone morphologies: type I clones have a smoother edge, a flatter cell morphology, and closer contact between cells; type II clones have a relatively irregular edge, a more uplifted cell morphology, and a more dispersed distribution among cells. Furthermore, it was reported that the type II clones grew faster (see Figure 2). Compared to dermal fibroblasts or adult adipose-derived mesenchymal stem cells, USCs demonstrate faster reprogramming kinetics, as well as higher efficiency [24, 29], which may result from the high telomerase activity observed in the endogenous expression reprogramming factors c-myc, Klf4, and USCs [29]. Therefore, identifying the source of stem cells is crucial since the process can generate a clear understanding of pluripotency as a potential application of urine cells.

## 3. Methods of Reprogramming Urine Cells

The methods used to isolate cells from urine reprogramming have evolved from integrated to nonintegrated. Since extracellular vectors are impossible to easily integrate into the cell genome, the use of a nonintegrated approach, compared to integrated, reduces the potential risks associated with the genomic integrity of hiPSCs cells [30, 31].

### 3.1. Integration

The first widely used reprogramming method involved the transfection of urine cells with retroviruses carrying the exogenous transcription factors. Here, urine cell isolation is performed immediately after urine collection in the clinics. Urine collected from an individual should produce enough urine cells to induce reprogramming after two weeks of culture and can be significantly reduced by pooling fresh urine cells from the same donor. Retroviral transfection with transcription factors occurs in about 3 to 4 weeks. Additionally, the iPSC generation process and yield of iPSC colonies are as high as 4%. Both in vivo and in vitro methods have shown that UiPSCs have the same pluripotency as ESCs and can continue to differentiate into three types of dermal tissues with satisfactory differentiation potential [28]. However, this method also integrates the exogenous genome into the host genome, increasing potential risks for the genomic integrity of hiPS cells, a typical example is the tumor formation caused by automatic activation of viral transgenes [32, 33]. Therefore, newer methods are required to impair this encounter.

### 3.2. Nonintegration

To solve the problem of gene integration caused by retrovirus reprogramming, researchers used the re-lox excision system [34] and piggyBac transposon-mediated gene transfer system [35] to eliminate integrated genes after gene expression. It is possible to remove the integrated gene, such as inserting–re-excision may interfere with the host cell genome and exert further effects. Therefore, investigators tend to use nonintegrated reprogramming methods. Among the existing strategies, the most widely used are episomal vector transfection [36] and nonintegrated virus-based methods, such as the adenovirus [37] and the Sendai virus [38] transduction and mRNA transfection [39]. These methods have shown promising reprogramming efficiency, although insufficient to produce 100% reprogramming cardiomyocytes.

#### 3.2.1. Episomal Vectors

Traditional methods were introduced owing to the safety concerns associated with viruses, with researchers reprogramming urine cells using pep4-eo2s-et2k, a nonintegrated episomal vector containing human genes OCT4, SOX2, SV40LT, and KLF4 [40]. In order to effectively improve the reprogramming efficiency, the transfection should contain miRNA cluster miR302-367 carrier. Moreover, urine cell agglutination was observed 8 days after transfection, transfection after 15 days, the iPSCs engraftment was observed, in a few classes, on the 20^th^ day, with typical iPSC colonies. Thus, the generated iPSCs are similar to ESCs, which demonstrate the potential of differentiation in in vivo and in vitro experiments and indicate that UiPSCs can continue into three layers of tissue. Rt-PCR showed that endogenous OCT4 was activated in iPSC colonies, and at the same time, exogenous transgenes (OCT4, SOX2, KLF4, SV40LT, and microRNA 302-367) were silenced. No free DNA was detected in iPSCs after 8 passages, which further indicated that iPSCs were not well integrated into the experiment [40]. Researchers have used free vectors to transfect transcription factors such as Oct4, Sox2, Lin28, l-myc, and Klf4 into urine cells, while added free vectors which mediated the EBNA1 gene expression, as well as a P53 knockout to improve reprogramming efficiency [24]. These methods eventually generated successful iPSCs.

#### 3.2.2. Adenovirus

Using nonintegrated adenovirus to express Oct4, Sox2, Klf4, and c-myc [37] briefly, researchers generated mouse iPSCs from fibroblasts and hepatocytes. Furthermore, HEK 293T cells were cotransfected with OKSM plasmid, psPAX2, and pMD2 to obtain the multipilocytic lentivirus vector encoding OKSM. Next, they are infected with USCs for reprogramming. After approximately 12 days, the colonies began to appear. iPSC colonies from USCs showed typical pluripotent stem cell morphology. In particular, various reactions are observed, such as the cells in the colonies were tightly arranged, the nucleoli of single cells were prominent, and also the ratio of the nucleus to the cytoplasm was increased. The expression of a group of multipotency-related genes was equivalent to that expressed by hESC H9, and at least three-fourths of mice injected with iPSC clones generated three types of embryonal teratomas [29].

#### 3.2.3. Sendai Virus

Reportedly, in 2009 [38] the Sendai virus (SeV), an RNA virus, did not increase the risk of altering the host genome, which constitutes an effective solution to produce safe iPSCs. SeV human iPSCs can express multipotent genes and also demonstrate demethylation characteristics of recombinant cells. Another study [29] reported that urine-derived renal epithelial cells, via infection with SeV-carrying Oct3/4, Sox2, Klf4, and c-myc gene UiPSCs, could induce reprogramming of UiPSCs. Recently, [41] it has been reported that SeV-infected with UCs contains human transcription factors (Oct4/Sox2, proto-oncogene, and Klf4), and typical UiPSC colonies appear around 3 to 4 weeks, similar in morphology to hESCs. The recombinant UiPSCs formed teratomas containing three dermal layers in mice, achieved via expressing hESC specific surface antigen. So far, SeV is the most promising method in the reprogramming of UiPSCs.

#### 3.2.4. Small Molecular Compounds

Although progress has been made in the generation of cardiomyocyte-like lineage from iPSCs, human iPSCs still face significant challenges in differentiating into functional cardiomyocytes [42]. The recent use of a variety of small molecular compounds has demonstrated that it is possible to improve the generation efficiency of iPSCs and reduces the reprogramming factors required during induction [43]. This suggests that certain reprogramming factors can be omitted [44, 45], which has also been applied in the reprogramming of urine cells.

#### 3.2.5. CRISPR Activation Technology

The CRISPR/Cas9 system can be designed by the combination of the sgRNA sequence with a specific purpose, while Cas9 sequences identify the sgRNA shear. Similarly, it has been observed with Cas9 and endonuclease after the deactivation of the dead Cas9 (dCas9), under the guidance of sgRNA, which can only be combined with the site, while the function of cutting is lost and dCas9 is reactivated. Notably, it has the potential to induce high expression of gene specificity. Researchers have observed that cell-induced pluripotency can be successfully achieved by targeting and activating endogenous Oct4 or Sox2 genes. Recently using the CRISPR activation technology, endogenous Oct4 or Sox2 genes in fibroblasts have been activated, and reprogramming towards pluripotency can trigger the formation of iPSCs with pluripotency. This not only provides a new approach for the generation of iPSCs but also affords new perspectives into the molecular mechanism of pluripotent induction [46]. It is believed that in the near future this method can also be used in the urine cell reprogramming process.

## 4. Application in Cardiovascular Disease

Existing studies have shown that iPSCs can be rapidly generated from human urine samples. The cells in urine can differentiate into beating cardiomyocytes by the same induction method as iPSCs from other sources. The primary objective in the treatment of cardiovascular diseases with human iPSCs is to simulate the physiology and overall function of the adult myocardium. Since most manifestations of cardiomyopathy occur in adulthood, it is necessary for the iPSCs to differentiate into mature CMs [47]. However, the application of UiPSCs in cardiovascular diseases is still limited. Only a few cardiovascular disease models have been established, and a huge research gap exists in the fields related to end-stage cardiovascular diseases and myocardial infarction [48].

### 4.1. Cardiomyocyte Models and Drug Study

Previously, studies on human genetic heart diseases and cardiac drug toxicity screening were mainly conducted in allogeneic systems or experimental animals. However, the traditional model is independent of the patient's genetic background, limiting the analysis and treatment of the disease. Moreover, they cannot adequately replicate human pathophysiological conditions. With the development of reprogramming technology, researchers have developed iPSCs from adult patient cells into human heart disease models, with different genetic backgrounds and disease phenotypes, demonstrating corresponding manifestations of cardiac dysfunction. Hence, iPSCs provide a high platform for the development of new drug therapies, as well as toxicological screening [49]. Based on the noninvasive nature of CMs derived from urine reprogramming to stem cells, many researchers have attempted to generate cardiac disease models from urine cells (see Table 1). However, owing to the constraints of heterogeneity and immature phenotype of iPSCs-CMs, it is necessary to generate results from experimental animals prior to concluding effectiveness [52].

#### 4.1.1. Long QT Syndrome Type 2

Jouni et al. [50] established a heart model of long QT syndrome type from a patient with HERG A561P gene mutation and his asymptomatic noncarrier mother's reprogrammed using the episomal vectors method. Furthermore, the asymptomatic noncarrier mother was also recombined [53]. This research used the matrix sandwich method to differentiate CMs cells from UhiPSCs. The reprogramming cells were shown to express atrial and ventricular cells, with ionic channels. Moreover, a comparison between the UhiPSCs-CMs from the patient with HERG and asymptomatic noncarrier mother revealed that the mutation resulted in transport defects and also reduced delayed rectification K+ current (IKr). The heart cells with type 2 long QT syndrome were examined, and the electrophysiological characteristics were established [50]. Cardiomyocytes were produced from somatic cells, demonstrating a complete set of the genetic background of the patient that could accurately express specific gene mutations. These findings suggest that UhiPSCs-CMs could be used as an in vitro model for regenerative medicine in many illnesses [41]. According to these findings, the hypothesis that UhiPSCs generate cardiomyocytes indicates a promising future in the treatment and cure of long QT syndrome.

#### 4.1.2. Duchenne Muscular Dystrophy

Cardiac myocytes were extracted from the urine of patients with Duchenne muscular dystrophy (DMD) by iPSCs. Through immunocytochemistry, RT-PCR, and teratoma formation, researchers confirmed the pluripotency of UiPSCs cloning. iPSCs clones from the urine of healthy volunteers and a DMD patient were also differentiated into beating cardiomyocytes, suggesting that cardiomyocytes effectively retained the dystrophy gene mutation in DMD patients. Physiological analyses indicated that myodystrophy in deficient cardiomyocytes demonstrated phenotypic differences from normal cardiomyocytes [29]. These results suggest the feasibility of generating cardiomyocytes from urine samples, with urine-derived cardiomyocytes retaining characteristics that may be used for further research on the mechanism and drug discovery.

#### 4.1.3. Dilated Cardiomyopathy (DCM)

Researchers have experimented with urine cells extracted from patients with dilated cardiomyopathy (DCM) and transfected with SeV-carrying Oct3/4, Sox2, Klf4, and c-myc genes. Based on the experiment, iPSCs were successfully generated. Since iPSCs carry disease-causing genes for specific diseases, the cytological basis was explored for further understanding of the pathogenesis, drug screening, and gene therapy of DCM [51].

#### 4.1.4. Ventricular Septal Defect

A ventricular septal defect (VSD) is a common type of congenital cardiac malformation. Volume overload that resulted from large volume VSDs may lead to heart failure (HF) [54]. The etiology of VSD-associated heart failure (HF) is complex, with increasing evidence emphasizing a genetic basis. The established UiPSCs cell line demonstrated a positive alkaline phosphatase activity, during which the RyR2 mutation was retained, and the expression of pluripotent markers indicated the possible differentiation of the three dermal layers in vivo. Notably, the differentiated myocardium could spontaneously contract, and the myocardial specific proteins and genes were strongly expressed during the reactions [55]. Compared with cardiomyocytes from H9 cells, autophagy was relatively high, suggesting that autophagy may be salient in the occurrence and development of VSD-associated HF. Furthermore, it indicates that UiPSCs obtained by this method can retain the genetic basis of the host and have the potential to be unique cell resources for molecular pathology research and HF treatment of VSD [41].

### 4.2. Prospects for the Application of Urine-Derived Induced Stem Cells

It is found that implantation of iPSCs can significantly improve cardiac function in the myocardial infarction model [56, 57]. However, this improvement usually occurs long before significant myogenesis [58]. Currently, the main mechanism of this cell therapy is paracrine, which includes the release of cytokines, chemokines, and growth factors, inhibition of apoptosis and fibrosis, enhancement of contractility, and activation of the endogenous regeneration mechanism [59, 60]. In addition to improving myocardial infarction, it demonstrated the ability to repair heart defects in animal experiments [61]. Hence, it may also be used to treat congenital heart defects. Besides, researchers have observed that hESC-CMs can act as a pacemaker in the pig heart with complete atrioventricular block and restore the mechanical and electrical properties of the myocardium [62]. Subsequent experiments that were conducted in guinea pigs also confirmed that hESC could be used for cardiac pacing [63]. iPSCs can also serve as biological pacemakers in patients with acquired heart disease, avoiding the clinical applications of implantable pacemakers, including infections and immune rejection. However, iPSCs still face many technical difficulties in clinical application, with an immediate and urgent need to further identify their tumorigenicity and immunogenicity and other related problems that occur later [64]. Therefore with the advantages of a urine cell, it is expected to become the original cell of the biological pacemaker.

Moreover, Ieda et al. successfully induced mouse cardiac fibroblasts into functional cardiomyocytes, with newer methods using GMT retrovirus for the first time [5]. In vivo studies also show that locally delivered retroviruses containing GMT can effectively reprogram fibroblasts, that reside in the heart of mice after coronary artery ligation, into CMs. Genetic pedigree tracing methods further observed that the induced CMs were derived from cardiac fibroblasts. Furthermore, three months after coronary artery ligation, the infarction area was significantly reduced, and cardiac function was improved in mice that used GMT [65]. More importantly, researchers have achieved the direct reprogramming of human fibroblasts into cardiomyocytes in a variety of methods [66]. This direct reprogramming bypasses the pluripotent phase, which can produce CMs more efficiently and significantly, reducing the tumorigenic risk associated with the usage of pluripotent stem cells. Although the direct reprogramming efficiency of human adult cells is relatively low at this stage, its advantages are worth researching for more suitable initial cell and differentiation conditions.

#### 4.2.1. Perspectives

iPSCs have significant advantages over ESCs. Mainly, iPSCs reprogram somatic cells, potentially facilitating in vitro reconstruction of patient-specific disease phenotypes. For example, CMs derived from hiPSCs is a new therapeutic strategy that might alter the future of cardiovascular treatment. Since iPSCs are relatively similar to ESCs, the traditional method can be used to perform CMs differentiation from hESCs and can also be used to induce the iPSCs differentiation process. Urine cells have become an ideal source of iPSCs due to their advantages of not requiring innovation and repeatability, and owing to their significantly higher reprogramming efficiency than dermal and mesenchymal stem cells, whether through retroviral infection or episomal vectors. Some scholars treat the successful differentiation of UiPSCs as a function of the myocardial cells. However, the differentiation of the myocardial cells still needs to be improved in terms of its maturity. Additionally, pluripotent cells with tumorigenicity cannot be directly applied to patients in the clinic. Therefore, the predifferentiation of the pluripotent cell into the target cell type is crucial before transplantation. New reprogramming techniques that avoid viral genome integration are available and the injection of differentiated cells can overcome significant challenges. However, reprogramming, expansion, differentiation, and CM purification protocols need to be optimized before the iPSC technology can be used clinically [31, 67].

## Figures and Tables

**Table 1:** Application of urine cell reprogramming in cardiovascular disease models.

Author	Year	Disease	Reprogramming	Typical colony	UiPSCs pluripotency	Cardiomyocyte induction	Functional cardiomyocytes		
							Efficiency	Spontaneously contractive	Reference
Guan et al.	2013	Duchenne muscular dystrophy	A polycistronic lentiviral vector Oct-3/4, Sox2, Klf4, and c-Myc	10–14 days	Teratoma with all 3 germ layers	Activin/BMP method	80%	After 8–20 days of differentiation	[29]	
Jouni et al.	2015	Type 2 long QT syndrome	Episomal vectors OCT3/4, SOX2, KLF4, MYC, LIN28, NANOG, SV40LT		Teratoma with all 3 germ layers	Matrix sandwich method	—	After 6–8 days of differentiation	[50]	
Lin et al.	2016	Dilated cardiomyopathy	Sendai virus OCT3/4, Sox2, Klf4, and c-Myc	—	Teratoma with all 3 germ layers	—	—	—	[51]	
Cao et al.	2018	Ventricular septal defect	Sendai virus sOct4/Sox2, c-Myc, Klf4	Around 3–4 weeks	Teratoma with all 3 germ layers	Small-molecule modulation of Wnt signaling	75–80%	After 12 days of differentiation	[41]	
